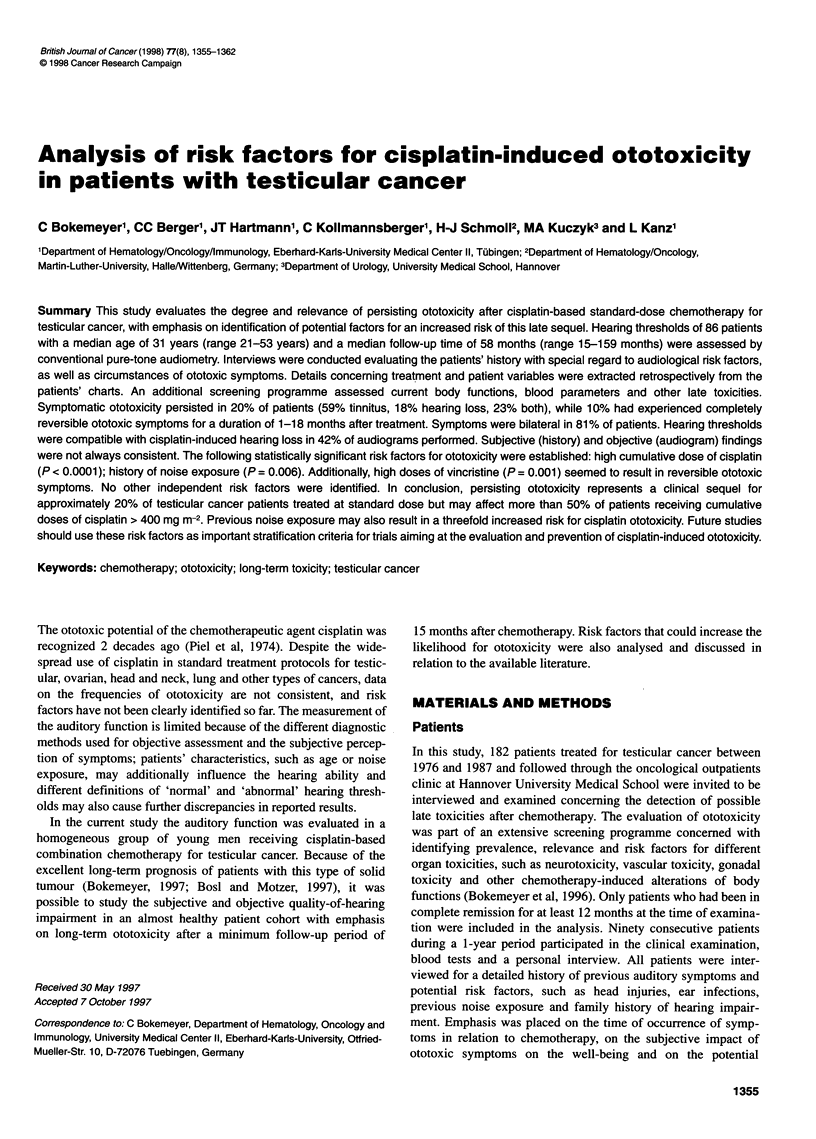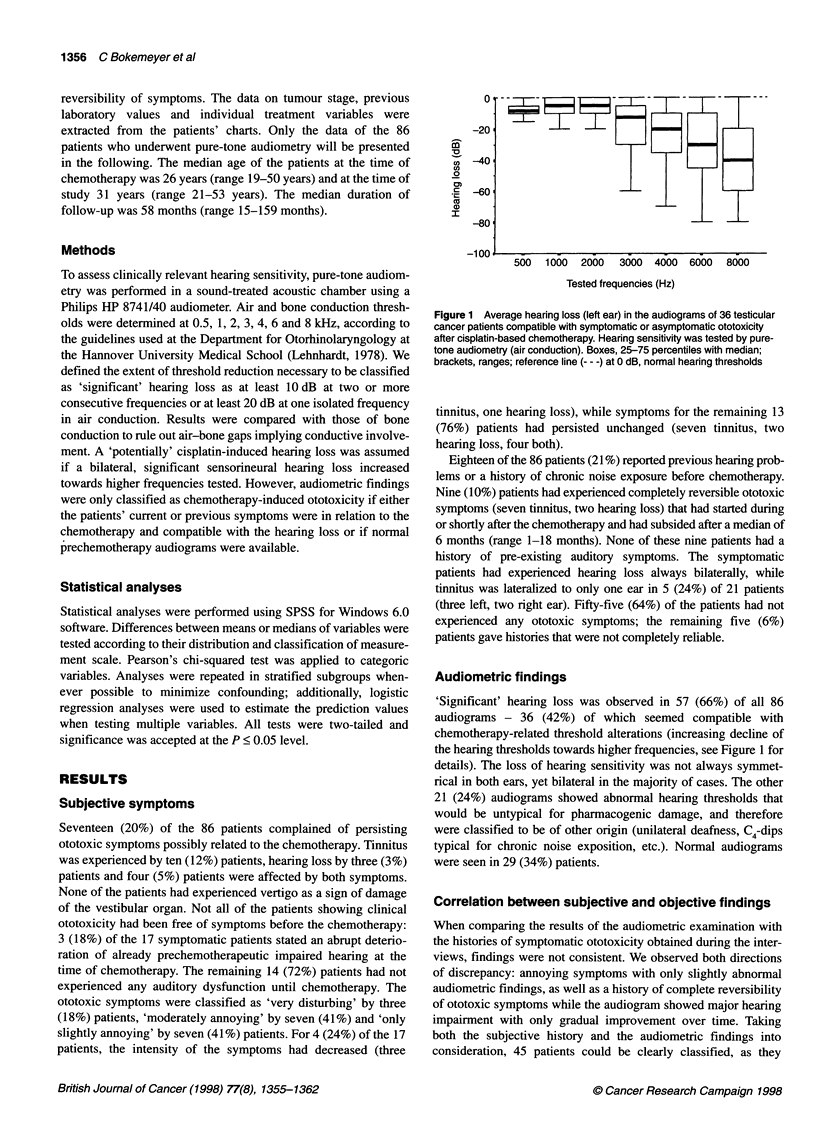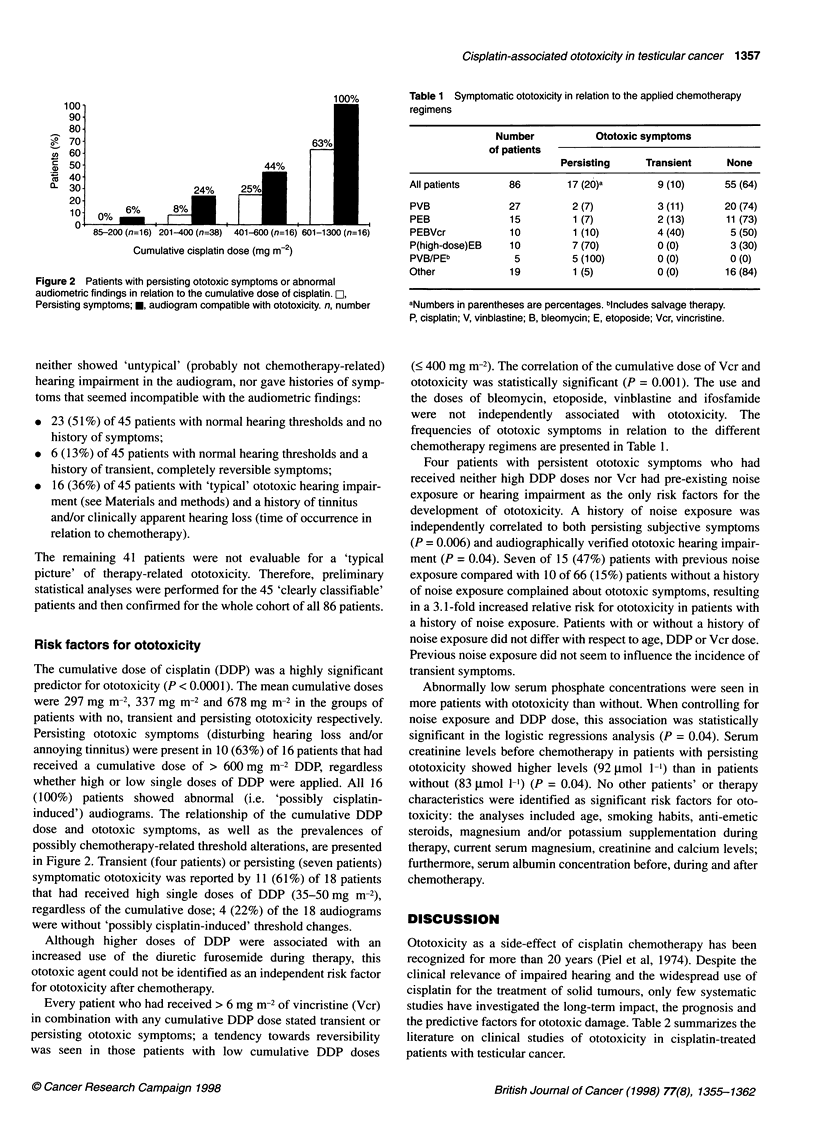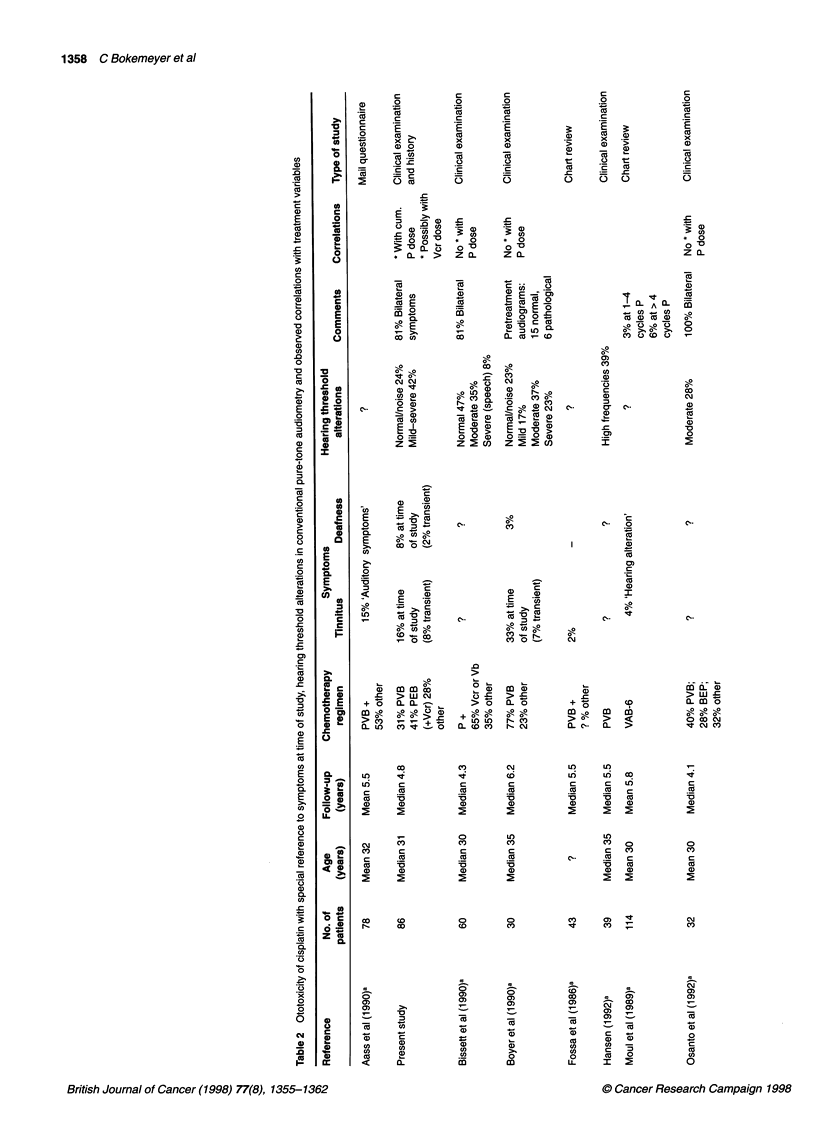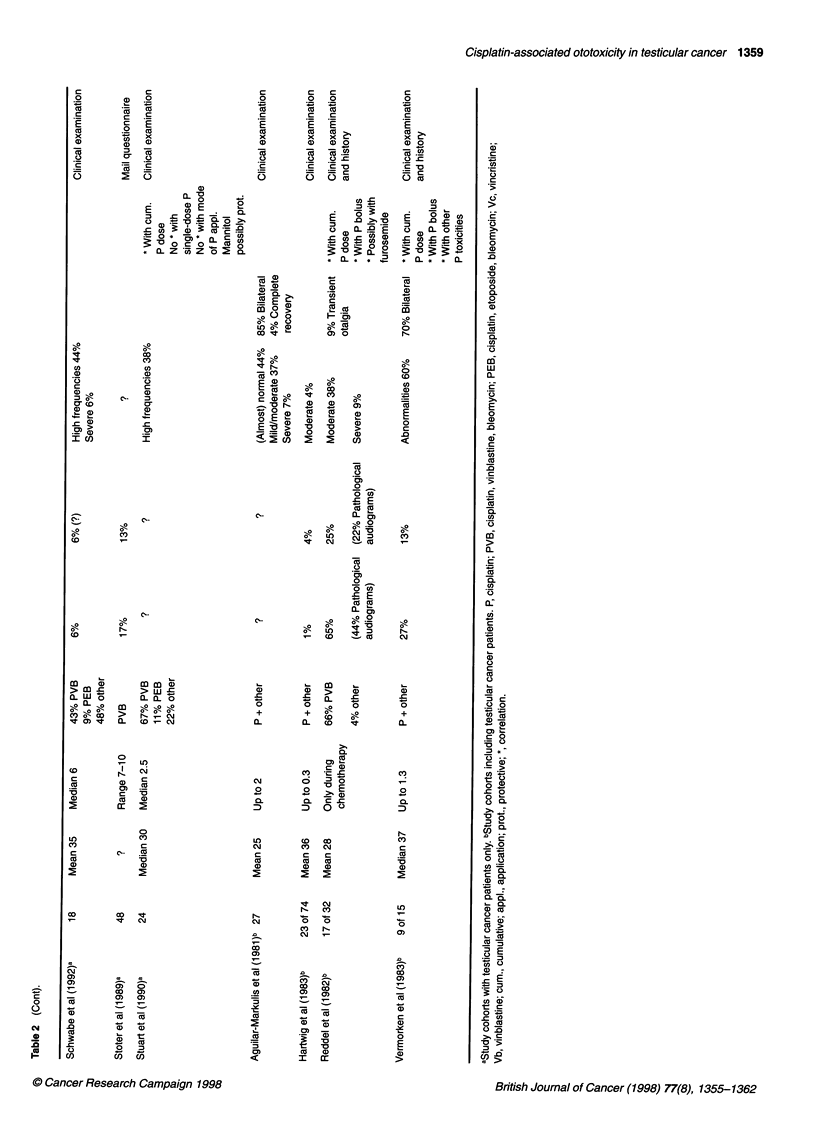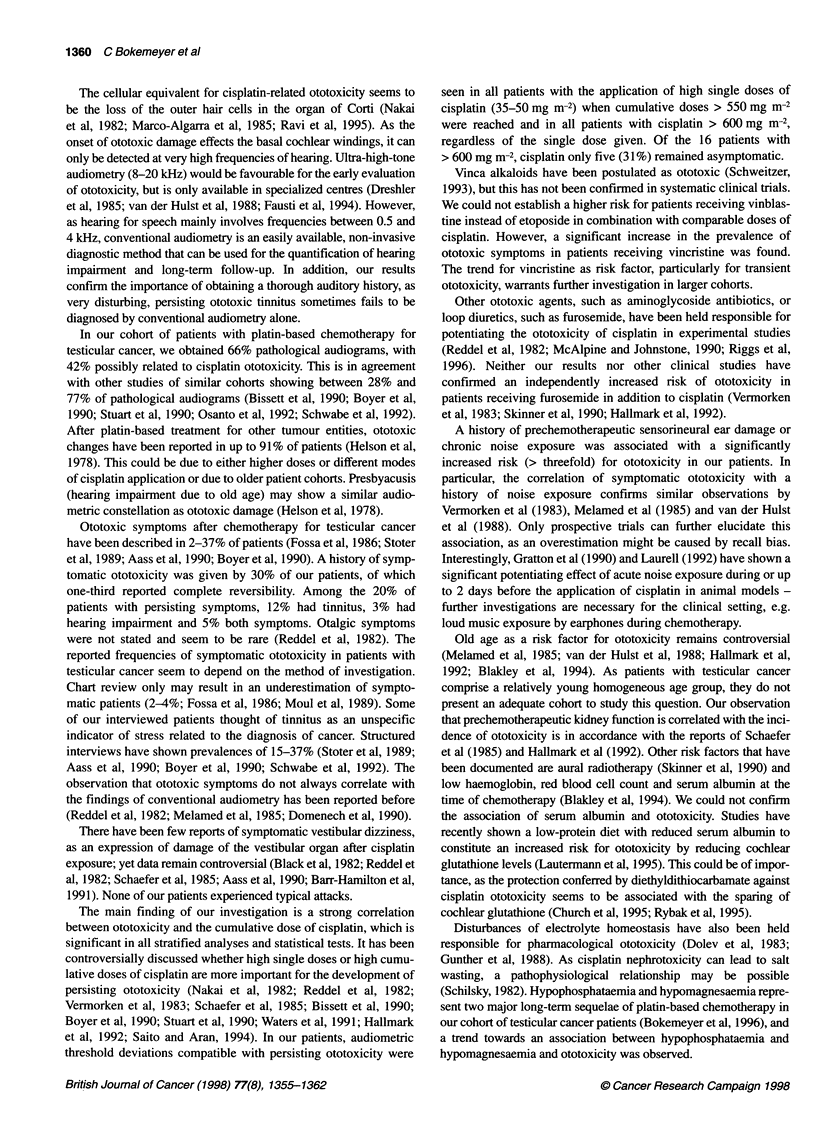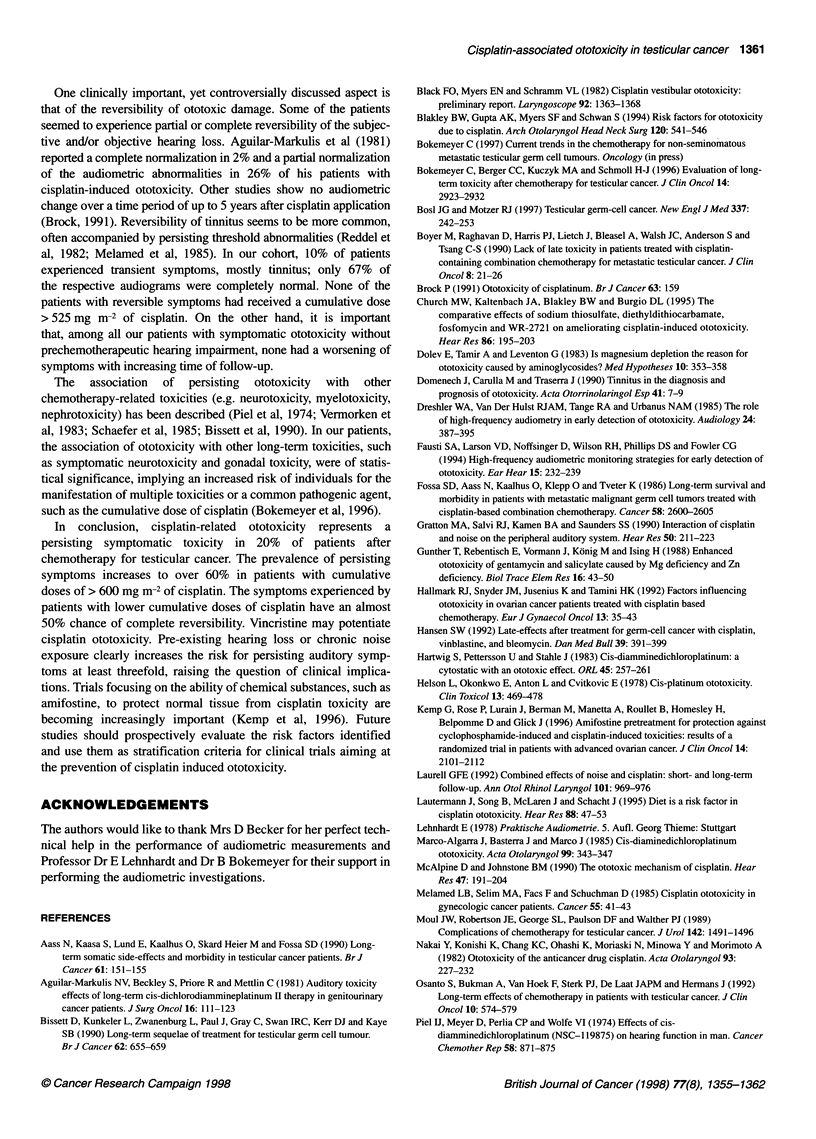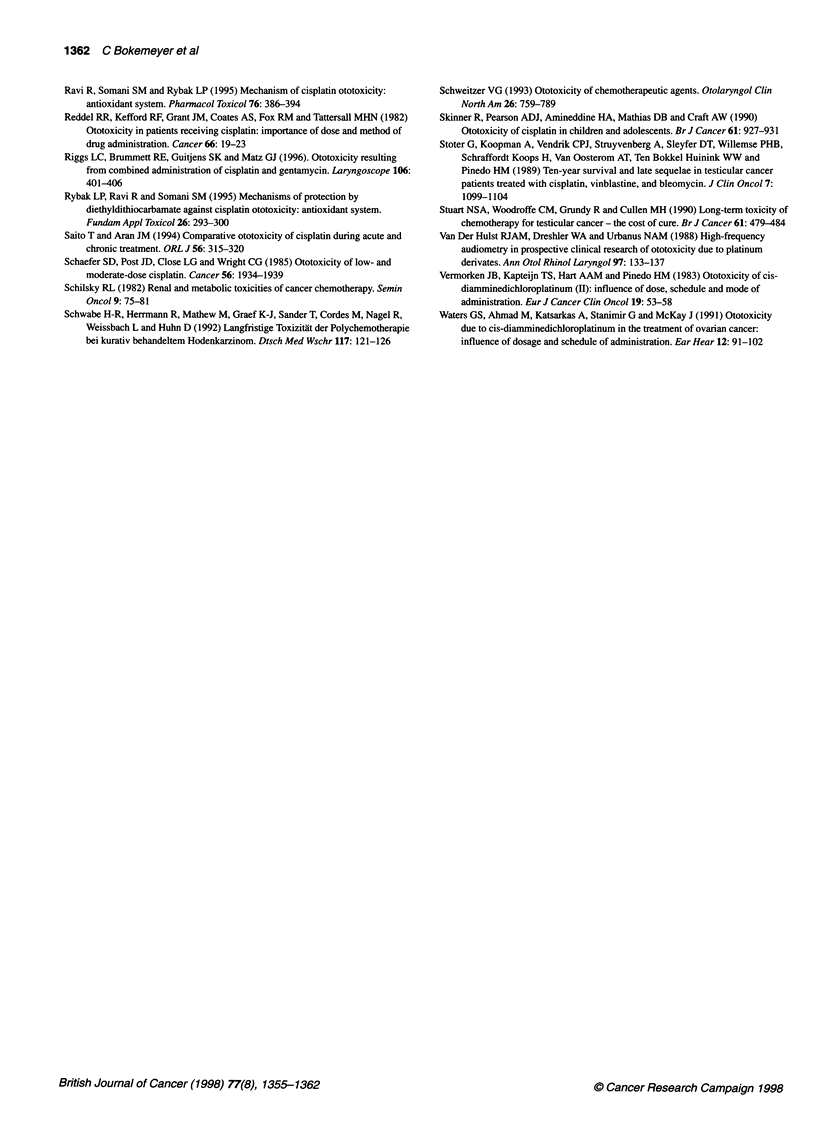# Analysis of risk factors for cisplatin-induced ototoxicity in patients with testicular cancer.

**DOI:** 10.1038/bjc.1998.226

**Published:** 1998-04

**Authors:** C. Bokemeyer, C. C. Berger, J. T. Hartmann, C. Kollmannsberger, H. J. Schmoll, M. A. Kuczyk, L. Kanz

**Affiliations:** Department of Hematology/Oncology/Immunology, Eberhard-Karls-University Medical Center II, TÃ¼bingen, Germany.

## Abstract

This study evaluates the degree and relevance of persisting ototoxicity after cisplatin-based standard-dose chemotherapy for testicular cancer, with emphasis on identification of potential factors for an increased risk of this late sequel. Hearing thresholds of 86 patients with a median age of 31 years (range 21-53 years) and a median follow-up time of 58 months (range 15-159 months) were assessed by conventional pure-tone audiometry. Interviews were conducted evaluating the patients' history with special regard to audiological risk factors, as well as circumstances of ototoxic symptoms. Details concerning treatment and patient variables were extracted retrospectively from the patients' charts. An additional screening programme assessed current body functions, blood parameters and other late toxicities. Symptomatic ototoxicity persisted in 20% of patients (59% tinnitus, 18% hearing loss, 23% both), while 10% had experienced completely reversible ototoxic symptoms for a duration of 1-18 months after treatment. Symptoms were bilateral in 81% of patients. Hearing thresholds were compatible with cisplatin-induced hearing loss in 42% of audiograms performed. Subjective (history) and objective (audiogram) findings were not always consistent. The following statistically significant risk factors for ototoxicity were established: high cumulative dose of cisplatin (P < 0.0001); history of noise exposure (P = 0.006). Additionally, high doses of vincristine (P = 0.001) seemed to result in reversible ototoxic symptoms. No other independent risk factors were identified. In conclusion, persisting ototoxicity represents a clinical sequel for approximately 20% of testicular cancer patients treated at standard dose but may affect more than 50% of patients receiving cumulative doses of cisplatin > 400 mg m(-2). Previous noise exposure may also result in a threefold increased risk for cisplatin ototoxicity. Future studies should use these risk factors as important stratification criteria for trials aiming at the evaluation and prevention of cisplatin-induced ototoxicity.


British Journal of Cancer (1998) 77(8), 1355–1362
© 1998 Cancer Research Campaign

### Analysis of risk factors for cisplatin-induced ototoxicity in patients with testicular cancer

C Bokemeyer[1], CC Berger[1], JT Hartmann[1], C Kollmannsberger[1], H-J Schmoll[2], MA Kuczyk[3] and L Kanz[1]

[1]Department of Hematology/Oncology/Immunology, Eberhard-Karls-University Medical Center II, Tübingen; [2]Department of Hematology/Oncology, Martin-Luther-University, Halle/Wittenberg, Germany; [3]Department of Urology, University Medical School, Hannover

**Summary** This study evaluates the degree and relevance of persisting ototoxicity after cisplatin-based standard-dose chemotherapy for testicular cancer, with emphasis on identification of potential factors for an increased risk of this late sequel. Hearing thresholds of 86 patients with a median age of 31 years (range 21–53 years) and a median follow-up time of 58 months (range 15–159 months) were assessed by conventional pure-tone audiometry. Interviews were conducted evaluating the patients' history with special regard to audiological risk factors, as well as circumstances of ototoxic symptoms. Details concerning treatment and patient variables were extracted retrospectively from the patients' charts. An additional screening programme assessed current body functions, blood parameters and other late toxicities. Symptomatic ototoxicity persisted in 20% of patients (59% tinnitus, 18% hearing loss, 23% both), while 10% had experienced completely reversible ototoxic symptoms for a duration of 1–18 months after treatment. Symptoms were bilateral in 81% of patients. Hearing thresholds were compatible with cisplatin-induced hearing loss in 42% of audiograms performed. Subjective (history) and objective (audiogram) findings were not always consistent. The following statistically significant risk factors for ototoxicity were established: high cumulative dose of cisplatin ($P < 0.0001$); history of noise exposure ($P = 0.006$). Additionally, high doses of vincristine ($P = 0.001$) seemed to result in reversible ototoxic symptoms. No other independent risk factors were identified. In conclusion, persisting ototoxicity represents a clinical sequel for approximately 20% of testicular cancer patients treated at standard dose but may affect more than 50% of patients receiving cumulative doses of cisplatin > 400 mg $m^{-2}$. Previous noise exposure may also result in a threefold increased risk for cisplatin ototoxicity. Future studies should use these risk factors as important stratification criteria for trials aiming at the evaluation and prevention of cisplatin-induced ototoxicity.

**Keywords:** chemotherapy; ototoxicity; long-term toxicity; testicular cancer

The ototoxic potential of the chemotherapeutic agent cisplatin was recognized 2 decades ago (Piel et al, 1974). Despite the widespread use of cisplatin in standard treatment protocols for testicular, ovarian, head and neck, lung and other types of cancers, data on the frequencies of ototoxicity are not consistent, and risk factors have not been clearly identified so far. The measurement of the auditory function is limited because of the different diagnostic methods used for objective assessment and the subjective perception of symptoms; patients' characteristics, such as age or noise exposure, may additionally influence the hearing ability and different definitions of 'normal' and 'abnormal' hearing thresholds may also cause further discrepancies in reported results.

In the current study the auditory function was evaluated in a homogeneous group of young men receiving cisplatin-based combination chemotherapy for testicular cancer. Because of the excellent long-term prognosis of patients with this type of solid tumour (Bokemeyer, 1997; Bosl and Motzer, 1997), it was possible to study the subjective and objective quality-of-hearing impairment in an almost healthy patient cohort with emphasis on long-term ototoxicity after a minimum follow-up period of 15 months after chemotherapy. Risk factors that could increase the likelihood for ototoxicity were also analysed and discussed in relation to the available literature.

*Received 30 May 1997*
*Accepted 7 October 1997*

*Correspondence to:* C Bokemeyer, Department of Hematology, Oncology and Immunology, University Medical Center II, Eberhard-Karls-University, Otfried-Mueller-Str. 10, D-72076 Tuebingen, Germany

### MATERIALS AND METHODS

### Patients

In this study, 182 patients treated for testicular cancer between 1976 and 1987 and followed through the oncological outpatients clinic at Hannover University Medical School were invited to be interviewed and examined concerning the detection of possible late toxicities after chemotherapy. The evaluation of ototoxicity was part of an extensive screening programme concerned with identifying prevalence, relevance and risk factors for different organ toxicities, such as neurotoxicity, vascular toxicity, gonadal toxicity and other chemotherapy-induced alterations of body functions (Bokemeyer et al, 1996). Only patients who had been in complete remission for at least 12 months at the time of examination were included in the analysis. Ninety consecutive patients during a 1-year period participated in the clinical examination, blood tests and a personal interview. All patients were interviewed for a detailed history of previous auditory symptoms and potential risk factors, such as head injuries, ear infections, previous noise exposure and family history of hearing impairment. Emphasis was placed on the time of occurrence of symptoms in relation to chemotherapy, on the subjective impact of ototoxic symptoms on the well-being and on the potential reversibility of symptoms. The data on tumour stage, previous laboratory values and individual treatment variables were extracted from the patients' charts. Only the data of the 86 patients who underwent pure-tone audiometry will be presented in the following. The median age of the patients at the time of chemotherapy was 26 years (range 19–50 years) and at the time of study 31 years (range 21–53 years). The median duration of follow-up was 58 months (range 15–159 months).

### Methods

To assess clinically relevant hearing sensitivity, pure-tone audiometry was performed in a sound-treated acoustic chamber using a Philips HP 8741/40 audiometer. Air and bone conduction thresholds were determined at 0.5, 1, 2, 3, 4, 6 and 8 kHz, according to the guidelines used at the Department for Otorhinolaryngology at the Hannover University Medical School (Lehnhardt, 1978). We defined the extent of threshold reduction necessary to be classified as 'significant' hearing loss as at least 10 dB at two or more consecutive frequencies or at least 20 dB at one isolated frequency in air conduction. Results were compared with those of bone conduction to rule out air–bone gaps implying conductive involvement. A 'potentially' cisplatin-induced hearing loss was assumed if a bilateral, significant sensorineural hearing loss increased towards higher frequencies tested. However, audiometric findings were only classified as chemotherapy-induced ototoxicity if either the patients' current or previous symptoms were in relation to the chemotherapy and compatible with the hearing loss or if normal prechemotherapy audiograms were available.

### Statistical analyses

Statistical analyses were performed using SPSS for Windows 6.0 software. Differences between means or medians of variables were tested according to their distribution and classification of measurement scale. Pearson's chi-squared test was applied to categoric variables. Analyses were repeated in stratified subgroups whenever possible to minimize confounding; additionally, logistic regression analyses were used to estimate the prediction values when testing multiple variables. All tests were two-tailed and significance was accepted at the $P \leq 0.05$ level.

### RESULTS

### Subjective symptoms

Seventeen (20%) of the 86 patients complained of persisting ototoxic symptoms possibly related to the chemotherapy. Tinnitus was experienced by ten (12%) patients, hearing loss by three (3%) patients and four (5%) patients were affected by both symptoms. None of the patients had experienced vertigo as a sign of damage of the vestibular organ. Not all of the patients showing clinical ototoxicity had been free of symptoms before the chemotherapy: 3 (18%) of the 17 symptomatic patients stated an abrupt deterioration of already prechemotherapeutic impaired hearing at the time of chemotherapy. The remaining 14 (72%) patients had not experienced any auditory dysfunction until chemotherapy. The ototoxic symptoms were classified as 'very disturbing' by three (18%) patients, 'moderately annoying' by seven (41%) and 'only slightly annoying' by seven (41%) patients. For 4 (24%) of the 17 patients, the intensity of the symptoms had decreased (three tinnitus, one hearing loss), while symptoms for the remaining 13 (76%) patients had persisted unchanged (seven tinnitus, two hearing loss, four both).

**Figure 1** Average hearing loss (left ear) in the audiograms of 36 testicular cancer patients compatible with symptomatic or asymptomatic ototoxicity after cisplatin-based chemotherapy. Hearing sensitivity was tested by pure-tone audiometry (air conduction). Boxes, 25–75 percentiles with median; brackets, ranges; reference line (- - -) at 0 dB, normal hearing thresholds

Eighteen of the 86 patients (21%) reported previous hearing problems or a history of chronic noise exposure before chemotherapy. Nine (10%) patients had experienced completely reversible ototoxic symptoms (seven tinnitus, two hearing loss) that had started during or shortly after the chemotherapy and had subsided after a median of 6 months (range 1–18 months). None of these nine patients had a history of pre-existing auditory symptoms. The symptomatic patients had experienced hearing loss always bilaterally, while tinnitus was lateralized to only one ear in 5 (24%) of 21 patients (three left, two right ear). Fifty-five (64%) of the patients had not experienced any ototoxic symptoms; the remaining five (6%) patients gave histories that were not completely reliable.

### Audiometric findings

'Significant' hearing loss was observed in 57 (66%) of all 86 audiograms – 36 (42%) of which seemed compatible with chemotherapy-related threshold alterations (increasing decline of the hearing thresholds towards higher frequencies, see Figure 1 for details). The loss of hearing sensitivity was not always symmetrical in both ears, yet bilateral in the majority of cases. The other 21 (24%) audiograms showed abnormal hearing thresholds that would be untypical for pharmacogenic damage, and therefore were classified to be of other origin (unilateral deafness, $C_4$-dips typical for chronic noise exposition, etc.). Normal audiograms were seen in 29 (34%) patients.

### Correlation between subjective and objective findings

When comparing the results of the audiometric examination with the histories of symptomatic ototoxicity obtained during the interviews, findings were not consistent. We observed both directions of discrepancy: annoying symptoms with only slightly abnormal audiometric findings, as well as a history of complete reversibility of ototoxic symptoms while the audiogram showed major hearing impairment with only gradual improvement over time. Taking both the subjective history and the audiometric findings into consideration, 45 patients could be clearly classified, as they

© Cancer Research Campaign 1998

**Figure 2** Patients with persisting ototoxic symptoms or abnormal audiometric findings in relation to the cumulative dose of cisplatin. □, Persisting symptoms; ■, audiogram compatible with ototoxicity. *n*, number

**Table 1** Symptomatic ototoxicity in relation to the applied chemotherapy regimens

| | Number of patients | Ototoxic symptoms | | |
|---|---|---|---|---|
| | | Persisting | Transient | None |
| All patients | 86 | 17 (20)[a] | 9 (10) | 55 (64) |
| PVB | 27 | 2 (7) | 3 (11) | 20 (74) |
| PEB | 15 | 1 (7) | 2 (13) | 11 (73) |
| PEBVcr | 10 | 1 (10) | 4 (40) | 5 (50) |
| P(high-dose)EB | 10 | 7 (70) | 0 (0) | 3 (30) |
| PVB/PE[b] | 5 | 5 (100) | 0 (0) | 0 (0) |
| Other | 19 | 1 (5) | 0 (0) | 16 (84) |

[a]Numbers in parentheses are percentages. [b]Includes salvage therapy.
P, cisplatin; V, vinblastine; B, bleomycin; E, etoposide; Vcr, vincristine.

neither showed 'untypical' (probably not chemotherapy-related) hearing impairment in the audiogram, nor gave histories of symptoms that seemed incompatible with the audiometric findings:

- 23 (51%) of 45 patients with normal hearing thresholds and no history of symptoms;
- 6 (13%) of 45 patients with normal hearing thresholds and a history of transient, completely reversible symptoms;
- 16 (36%) of 45 patients with 'typical' ototoxic hearing impairment (see Materials and methods) and a history of tinnitus and/or clinically apparent hearing loss (time of occurrence in relation to chemotherapy).

The remaining 41 patients were not evaluable for a 'typical picture' of therapy-related ototoxicity. Therefore, preliminary statistical analyses were performed for the 45 'clearly classifiable' patients and then confirmed for the whole cohort of all 86 patients.

### Risk factors for ototoxicity

The cumulative dose of cisplatin (DDP) was a highly significant predictor for ototoxicity ($P < 0.0001$). The mean cumulative doses were 297 mg $m^{-2}$, 337 mg $m^{-2}$ and 678 mg $m^{-2}$ in the groups of patients with no, transient and persisting ototoxicity respectively. Persisting ototoxic symptoms (disturbing hearing loss and/or annoying tinnitus) were present in 10 (63%) of 16 patients that had received a cumulative dose of > 600 mg $m^{-2}$ DDP, regardless whether high or low single doses of DDP were applied. All 16 (100%) patients showed abnormal (i.e. 'possibly cisplatin-induced') audiograms. The relationship of the cumulative DDP dose and ototoxic symptoms, as well as the prevalences of possibly chemotherapy-related threshold alterations, are presented in Figure 2. Transient (four patients) or persisting (seven patients) symptomatic ototoxicity was reported by 11 (61%) of 18 patients that had received high single doses of DDP (35–50 mg $m^{-2}$), regardless of the cumulative dose; 4 (22%) of the 18 audiograms were without 'possibly cisplatin-induced' threshold changes.

Although higher doses of DDP were associated with an increased use of the diuretic furosemide during therapy, this ototoxic agent could not be identified as an independent risk factor for ototoxicity after chemotherapy.

Every patient who had received > 6 mg $m^{-2}$ of vincristine (Vcr) in combination with any cumulative DDP dose stated transient or persisting ototoxic symptoms; a tendency towards reversibility was seen in those patients with low cumulative DDP doses (≤ 400 mg $m^{-2}$). The correlation of the cumulative dose of Vcr and ototoxicity was statistically significant ($P = 0.001$). The use and the doses of bleomycin, etoposide, vinblastine and ifosfamide were not independently associated with ototoxicity. The frequencies of ototoxic symptoms in relation to the different chemotherapy regimens are presented in Table 1.

Four patients with persistent ototoxic symptoms who had received neither high DDP doses nor Vcr had pre-existing noise exposure or hearing impairment as the only risk factors for the development of ototoxicity. A history of noise exposure was independently correlated to both persisting subjective symptoms ($P = 0.006$) and audiographically verified ototoxic hearing impairment ($P = 0.04$). Seven of 15 (47%) patients with previous noise exposure compared with 10 of 66 (15%) patients without a history of noise exposure complained about ototoxic symptoms, resulting in a 3.1-fold increased relative risk for ototoxicity in patients with a history of noise exposure. Patients with or without a history of noise exposure did not differ with respect to age, DDP or Vcr dose. Previous noise exposure did not seem to influence the incidence of transient symptoms.

Abnormally low serum phosphate concentrations were seen in more patients with ototoxicity than without. When controlling for noise exposure and DDP dose, this association was statistically significant in the logistic regressions analysis ($P = 0.04$). Serum creatinine levels before chemotherapy in patients with persisting ototoxicity showed higher levels (92 μmol $l^{-1}$) than in patients without (83 μmol $l^{-1}$) ($P = 0.04$). No other patients' or therapy characteristics were identified as significant risk factors for ototoxicity: the analyses included age, smoking habits, anti-emetic steroids, magnesium and/or potassium supplementation during therapy, current serum magnesium, creatinine and calcium levels; furthermore, serum albumin concentration before, during and after chemotherapy.

### DISCUSSION

Ototoxicity as a side-effect of cisplatin chemotherapy has been recognized for more than 20 years (Piel et al, 1974). Despite the clinical relevance of impaired hearing and the widespread use of cisplatin for the treatment of solid tumours, only few systematic studies have investigated the long-term impact, the prognosis and the predictive factors for ototoxic damage. Table 2 summarizes the literature on clinical studies of ototoxicity in cisplatin-treated patients with testicular cancer.

© Cancer Research Campaign 1998

Table 2 Ototoxicity of cisplatin with special reference to symptoms at time of study, hearing threshold alterations in conventional pure-tone audiometry and observed correlations with treatment variables

| Reference | No. of patients | Age (years) | Follow-up (years) | Chemotherapy regimen | Symptoms Tinnitus | Symptoms Deafness | Hearing threshold alterations | Comments | Correlations | Type of study |
|---|---|---|---|---|---|---|---|---|---|---|
| Aass et al (1990)[a] | 78 | Mean 32 | Mean 5.5 | PVB + 53% other | 15% 'Auditory symptoms' | | ? | | | Mail questionnaire |
| Present study | 86 | Median 31 | Median 4.8 | 31% PVB 41% PEB (+Vcr) 28% other | 16% at time of study (8% transient) | 8% at time of study (2% transient) | Normal/noise 24% Mild–severe 42% | 81% Bilateral symptoms | * With cum. P dose * Possibly with Vcr dose | Clinical examination and history |
| Bissett et al (1990)[a] | 60 | Median 30 | Median 4.3 | P + 65% Vcr or Vb 35% other | ? | ? | Normal 47% Moderate 35% Severe (speech) 8% | 81% Bilateral | No * with P dose | Clinical examination |
| Boyer et al (1990)[a] | 30 | Median 35 | Median 6.2 | 77% PVB 23% other | 33% at time of study (7% transient) | 3% | Normal/noise 23% Mild 17% Moderate 37% Severe 23% | Pretreatment audiograms: 15 normal, 6 pathological | No * with P dose | Clinical examination |
| Fossa et al (1986)[a] | 43 | ? | Median 5.5 | PVB + ? % other | 2% | – | ? | | | Chart review |
| Hansen (1992)[a] | 39 | Median 35 | Median 5.5 | PVB | ? | ? | High frequencies 39% | | | Clinical examination |
| Moul et al (1989)[a] | 114 | Mean 30 | Mean 5.8 | VAB-6 | 4% 'Hearing alteration' | | ? | 3% at 1–4 cycles P 6% at > 4 cycles P | | Chart review |
| Osanto et al (1992)[a] | 32 | Mean 30 | Median 4.1 | 40% PVB; 28% BEP; 32% other | ? | ? | Moderate 28% | 100% Bilateral | No * with P dose | Clinical examination |

© Cancer Research Campaign 1998

**Table 2** (Cont).

| | | | | | | | | | | |
|---|---|---|---|---|---|---|---|---|---|---|
| Schwabe et al (1992)[a] | 18 | Mean 35 | Median 6 | 43% PVB<br>9% PEB<br>48% other | 6% | 6% (?) | High frequencies 44%<br>Severe 6% | | | Clinical examination |
| Stoter et al (1989)[a] | 48 | ? | Range 7–10 | PVB | 17% | 13% | ? | | | Mail questionnaire |
| Stuart et al (1990)[a] | 24 | Median 30 | Median 2.5 | 67% PVB<br>11% PEB<br>22% other | ? | ? | High frequencies 38% | | * With cum. P dose<br>No * with single-dose P<br>No * with mode of P appl.<br>Mannitol possibly prot. | Clinical examination |
| Aguilar-Markulis et al (1981)[b] | 27 | Mean 25 | Up to 2 | P + other | ? | ? | (Almost) normal 44%<br>Mild/moderate 37%<br>Severe 7% | 85% Bilateral<br>4% Complete recovery | | Clinical examination |
| Hartwig et al (1983)[b] | 23 of 74 | Mean 36 | Up to 0.3 | P + other | 1% | 4% | Moderate 4% | | | Clinical examination |
| Reddel et al (1982)[b] | 17 of 32 | Mean 28 | Only during chemotherapy | 66% PVB<br>4% other | 65%<br>(44% Pathological audiograms) | 25%<br>(22% Pathological audiograms) | Moderate 38%<br>Severe 9% | 9% Transient otalgia | * With cum. P dose<br>* With P bolus<br>* Possibly with furosemide | Clinical examination and history |
| Vermorken et al (1983)[b] | 9 of 15 | Median 37 | Up to 1.3 | P + other | 27% | 13% | Abnormalities 60% | 70% Bilateral | * With cum. P dose<br>* With P bolus<br>* With other P toxicities | Clinical examination and history |

[a]Study cohorts with testicular cancer patients only. [b]Study cohorts including testicular cancer patients. P, cisplatin; PVB, cisplatin, vinblastine, bleomycin; PEB, cisplatin, etoposide, bleomycin; Vc, vincristine; Vb, vinblastine; cum., cumulative; appl., application; prot., protective; *, correlation.

© Cancer Research Campaign 1998

The cellular equivalent for cisplatin-related ototoxicity seems to be the loss of the outer hair cells in the organ of Corti (Nakai et al, 1982; Marco-Algarra et al, 1985; Ravi et al, 1995). As the onset of ototoxic damage effects the basal cochlear windings, it can only be detected at very high frequencies of hearing. Ultra-high-tone audiometry (8–20 kHz) would be favourable for the early evaluation of ototoxicity, but is only available in specialized centres (Dreshler et al, 1985; van der Hulst et al, 1988; Fausti et al, 1994). However, as hearing for speech mainly involves frequencies between 0.5 and 4 kHz, conventional audiometry is an easily available, non-invasive diagnostic method that can be used for the quantification of hearing impairment and long-term follow-up. In addition, our results confirm the importance of obtaining a thorough auditory history, as very disturbing, persisting ototoxic tinnitus sometimes fails to be diagnosed by conventional audiometry alone.

In our cohort of patients with platin-based chemotherapy for testicular cancer, we obtained 66% pathological audiograms, with 42% possibly related to cisplatin ototoxicity. This is in agreement with other studies of similar cohorts showing between 28% and 77% of pathological audiograms (Bissett et al, 1990; Boyer et al, 1990; Stuart et al, 1990; Osanto et al, 1992; Schwabe et al, 1992). After platin-based treatment for other tumour entities, ototoxic changes have been reported in up to 91% of patients (Helson et al, 1978). This could be due to either higher doses or different modes of cisplatin application or due to older patient cohorts. Presbyacusis (hearing impairment due to old age) may show a similar audiometric constellation as ototoxic damage (Helson et al, 1978).

Ototoxic symptoms after chemotherapy for testicular cancer have been described in 2–37% of patients (Fossa et al, 1986; Stoter et al, 1989; Aass et al, 1990; Boyer et al, 1990). A history of symptomatic ototoxicity was given by 30% of our patients, of which one-third reported complete reversibility. Among the 20% of patients with persisting symptoms, 12% had tinnitus, 3% had hearing impairment and 5% both symptoms. Otalgic symptoms were not stated and seem to be rare (Reddel et al, 1982). The reported frequencies of symptomatic ototoxicity in patients with testicular cancer seem to depend on the method of investigation. Chart review only may result in an underestimation of symptomatic patients (2–4%; Fossa et al, 1986; Moul et al, 1989). Some of our interviewed patients thought of tinnitus as an unspecific indicator of stress related to the diagnosis of cancer. Structured interviews have shown prevalences of 15–37% (Stoter et al, 1989; Aass et al, 1990; Boyer et al, 1990; Schwabe et al, 1992). The observation that ototoxic symptoms do not always correlate with the findings of conventional audiometry has been reported before (Reddel et al, 1982; Melamed et al, 1985; Domenech et al, 1990).

There have been few reports of symptomatic vestibular dizziness, as an expression of damage of the vestibular organ after cisplatin exposure; yet data remain controversial (Black et al, 1982; Reddel et al, 1982; Schaefer et al, 1985; Aass et al, 1990; Barr-Hamilton et al, 1991). None of our patients experienced typical attacks.

The main finding of our investigation is a strong correlation between ototoxicity and the cumulative dose of cisplatin, which is significant in all stratified analyses and statistical tests. It has been controversially discussed whether high single doses or high cumulative doses of cisplatin are more important for the development of persisting ototoxicity (Nakai et al, 1982; Reddel et al, 1982; Vermorken et al, 1983; Schaefer et al, 1985; Bissett et al, 1990; Boyer et al, 1990; Stuart et al, 1990; Waters et al, 1991; Hallmark et al, 1992; Saito and Aran, 1994). In our patients, audiometric threshold deviations compatible with persisting ototoxicity were seen in all patients with the application of high single doses of cisplatin (35–50 mg $m^{-2}$) when cumulative doses > 550 mg $m^{-2}$ were reached and in all patients with cisplatin > 600 mg $m^{-2}$, regardless of the single dose given. Of the 16 patients with > 600 mg $m^{-2}$, cisplatin only five (31%) remained asymptomatic.

Vinca alkaloids have been postulated as ototoxic (Schweitzer, 1993), but this has not been confirmed in systematic clinical trials. We could not establish a higher risk for patients receiving vinblastine instead of etoposide in combination with comparable doses of cisplatin. However, a significant increase in the prevalence of ototoxic symptoms in patients receiving vincristine was found. The trend for vincristine as risk factor, particularly for transient ototoxicity, warrants further investigation in larger cohorts.

Other ototoxic agents, such as aminoglycoside antibiotics, or loop diuretics, such as furosemide, have been held responsible for potentiating the ototoxicity of cisplatin in experimental studies (Reddel et al, 1982; McAlpine and Johnstone, 1990; Riggs et al, 1996). Neither our results nor other clinical studies have confirmed an independently increased risk of ototoxicity in patients receiving furosemide in addition to cisplatin (Vermorken et al, 1983; Skinner et al, 1990; Hallmark et al, 1992).

A history of prechemotherapeutic sensorineural ear damage or chronic noise exposure was associated with a significantly increased risk (> threefold) for ototoxicity in our patients. In particular, the correlation of symptomatic ototoxicity with a history of noise exposure confirms similar observations by Vermorken et al (1983), Melamed et al (1985) and van der Hulst et al (1988). Only prospective trials can further elucidate this association, as an overestimation might be caused by recall bias. Interestingly, Gratton et al (1990) and Laurell (1992) have shown a significant potentiating effect of acute noise exposure during or up to 2 days before the application of cisplatin in animal models – further investigations are necessary for the clinical setting, e.g. loud music exposure by earphones during chemotherapy.

Old age as a risk factor for ototoxicity remains controversial (Melamed et al, 1985; van der Hulst et al, 1988; Hallmark et al, 1992; Blakley et al, 1994). As patients with testicular cancer comprise a relatively young homogeneous age group, they do not present an adequate cohort to study this question. Our observation that prechemotherapeutic kidney function is correlated with the incidence of ototoxicity is in accordance with the reports of Schaefer et al (1985) and Hallmark et al (1992). Other risk factors that have been documented are aural radiotherapy (Skinner et al, 1990) and low haemoglobin, red blood cell count and serum albumin at the time of chemotherapy (Blakley et al, 1994). We could not confirm the association of serum albumin and ototoxicity. Studies have recently shown a low-protein diet with reduced serum albumin to constitute an increased risk for ototoxicity by reducing cochlear glutathione levels (Lautermann et al, 1995). This could be of importance, as the protection conferred by diethyldithiocarbamate against cisplatin ototoxicity seems to be associated with the sparing of cochlear glutathione (Church et al, 1995; Rybak et al, 1995).

Disturbances of electrolyte homeostasis have also been held responsible for pharmacological ototoxicity (Dolev et al, 1983; Gunther et al, 1988). As cisplatin nephrotoxicity can lead to salt wasting, a pathophysiological relationship may be possible (Schilsky, 1982). Hypophosphataemia and hypomagnesaemia represent two major long-term sequelae of platin-based chemotherapy in our cohort of testicular cancer patients (Bokemeyer et al, 1996), and a trend towards an association between hypophosphataemia and hypomagnesaemia and ototoxicity was observed.

 © Cancer Research Campaign 1998

One clinically important, yet controversially discussed aspect is that of the reversibility of ototoxic damage. Some of the patients seemed to experience partial or complete reversibility of the subjective and/or objective hearing loss. Aguilar-Markulis et al (1981) reported a complete normalization in 2% and a partial normalization of the audiometric abnormalities in 26% of his patients with cisplatin-induced ototoxicity. Other studies show no audiometric change over a time period of up to 5 years after cisplatin application (Brock, 1991). Reversibility of tinnitus seems to be more common, often accompanied by persisting threshold abnormalities (Reddel et al, 1982; Melamed et al, 1985). In our cohort, 10% of patients experienced transient symptoms, mostly tinnitus; only 67% of the respective audiograms were completely normal. None of the patients with reversible symptoms had received a cumulative dose > 525 mg $m^{-2}$ of cisplatin. On the other hand, it is important that, among all our patients with symptomatic ototoxicity without prechemotherapeutic hearing impairment, none had a worsening of symptoms with increasing time of follow-up.

The association of persisting ototoxicity with other chemotherapy-related toxicities (e.g. neurotoxicity, myelotoxicity, nephrotoxicity) has been described (Piel et al, 1974; Vermorken et al, 1983; Schaefer et al, 1985; Bissett et al, 1990). In our patients, the association of ototoxicity with other long-term toxicities, such as symptomatic neurotoxicity and gonadal toxicity, were of statistical significance, implying an increased risk of individuals for the manifestation of multiple toxicities or a common pathogenic agent, such as the cumulative dose of cisplatin (Bokemeyer et al, 1996).

In conclusion, cisplatin-related ototoxicity represents a persisting symptomatic toxicity in 20% of patients after chemotherapy for testicular cancer. The prevalence of persisting symptoms increases to over 60% in patients with cumulative doses of > 600 mg $m^{-2}$ of cisplatin. The symptoms experienced by patients with lower cumulative doses of cisplatin have an almost 50% chance of complete reversibility. Vincristine may potentiate cisplatin ototoxicity. Pre-existing hearing loss or chronic noise exposure clearly increases the risk for persisting auditory symptoms at least threefold, raising the question of clinical implications. Trials focusing on the ability of chemical substances, such as amifostine, to protect normal tissue from cisplatin toxicity are becoming increasingly important (Kemp et al, 1996). Future studies should prospectively evaluate the risk factors identified and use them as stratification criteria for clinical trials aiming at the prevention of cisplatin induced ototoxicity.

### ACKNOWLEDGEMENTS

The authors would like to thank Mrs D Becker for her perfect technical help in the performance of audiometric measurements and Professor Dr E Lehnhardt and Dr B Bokemeyer for their support in performing the audiometric investigations.

### REFERENCES

Aass N, Kaasa S, Lund E, Kaalhus O, Skard Heier M and Fossa SD (1990) Long-term somatic side-effects and morbidity in testicular cancer patients. *Br J Cancer* **61**: 151–155

Aguilar-Markulis NV, Beckley S, Priore R and Mettlin C (1981) Auditory toxicity effects of long-term cis-dichlorodiammineplatinum II therapy in genitourinary cancer patients. *J Surg Oncol* **16**: 111–123

Bissett D, Kunkeler L, Zwanenburg L, Paul J, Gray C, Swan IRC, Kerr DJ and Kaye SB (1990) Long-term sequelae of treatment for testicular germ cell tumour. *Br J Cancer* **62**: 655–659

Black FO, Myers EN and Schramm VL (1982) Cisplatin vestibular ototoxicity: preliminary report. *Laryngoscope* **92**: 1363–1368

Blakley BW, Gupta AK, Myers SF and Schwan S (1994) Risk factors for ototoxicity due to cisplatin. *Arch Otolaryngol Head Neck Surg* **120**: 541–546

Bokemeyer C (1997) Current trends in the chemotherapy for non-seminomatous metastatic testicular germ cell tumours. *Oncology* (in press)

Bokemeyer C, Berger CC, Kuczyk MA and Schmoll H-J (1996) Evaluation of long-term toxicity after chemotherapy for testicular cancer. *J Clin Oncol* **14**: 2923–2932

Bosl JG and Motzer RJ (1997) Testicular germ-cell cancer. *New Engl J Med* **337**: 242–253

Boyer M, Raghavan D, Harris PJ, Lietch J, Bleasel A, Walsh JC, Anderson S and Tsang C-S (1990) Lack of late toxicity in patients treated with cisplatin-containing combination chemotherapy for metastatic testicular cancer. *J Clin Oncol* **8**: 21–26

Brock P (1991) Ototoxicity of cisplatinum. *Br J Cancer* **63**: 159

Church MW, Kaltenbach JA, Blakley BW and Burgio DL (1995) The comparative effects of sodium thiosulfate, diethyldithiocarbamate, fosfomycin and WR-2721 on ameliorating cisplatin-induced ototoxicity. *Hear Res* **86**: 195–203

Dolev E, Tamir A and Leventon G (1983) Is magnesium depletion the reason for ototoxicity caused by aminoglycosides? *Med Hypotheses* **10**: 353–358

Domenech J, Carulla M and Traserra J (1990) Tinnitus in the diagnosis and prognosis of ototoxicity. *Acta Otorrinolaringol Esp* **41**: 7–9

Dreshler WA, Van Der Hulst RJAM, Tange RA and Urbanus NAM (1985) The role of high-frequency audiometry in early detection of ototoxicity. *Audiology* **24**: 387–395

Fausti SA, Larson VD, Noffsinger D, Wilson RH, Phillips DS and Fowler CG (1994) High-frequency audiometric monitoring strategies for early detection of ototoxicity. *Ear Hear* **15**: 232–239

Fossa SD, Aass N, Kaalhus O, Klepp O and Tveter K (1986) Long-term survival and morbidity in patients with metastatic malignant germ cell tumors treated with cisplatin-based combination chemotherapy. *Cancer* **58**: 2600–2605

Gratton MA, Salvi RJ, Kamen BA and Saunders SS (1990) Interaction of cisplatin and noise on the peripheral auditory system. *Hear Res* **50**: 211–223

Gunther T, Rebentisch E, Vormann J, König M and Ising H (1988) Enhanced ototoxicity of gentamycin and salicylate caused by Mg deficiency and Zn deficiency. *Biol Trace Elem Res* **16**: 43–50

Hallmark RJ, Snyder JM, Jusenius K and Tamini HK (1992) Factors influencing ototoxicity in ovarian cancer patients treated with cisplatin based chemotherapy. *Eur J Gynaecol Oncol* **13**: 35–43

Hansen SW (1992) Late-effects after treatment for germ-cell cancer with cisplatin, vinblastine, and bleomycin. *Dan Med Bull* **39**: 391–399

Hartwig S, Pettersson U and Stahle J (1983) Cis-diamminedichloroplatinum: a cytostatic with an ototoxic effect. *ORL* **45**: 257–261

Helson L, Okonkwo E, Anton L and Cvitkovic E (1978) Cis-platinum ototoxicity. *Clin Toxicol* **13**: 469–478

Kemp G, Rose P, Lurain J, Berman M, Manetta A, Roullet B, Homesley H, Belpomme D and Glick J (1996) Amifostine pretreatment for protection against cyclophosphamide-induced and cisplatin-induced toxicities: results of a randomized trial in patients with advanced ovarian cancer. *J Clin Oncol* **14**: 2101–2112

Laurell GFE (1992) Combined effects of noise and cisplatin: short- and long-term follow-up. *Ann Otol Rhinol Laryngol* **101**: 969–976

Lautermann J, Song B, McLaren J and Schacht J (1995) Diet is a risk factor in cisplatin ototoxicity. *Hear Res* **88**: 47–53

Lehnhardt E (1978) *Praktische Audiometrie*. 5. Aufl. Georg Thieme: Stuttgart

Marco-Algarra J, Basterra J and Marco J (1985) Cis-diaminedichloroplatinum ototoxicity. *Acta Otolaryngol* **99**: 343–347

McAlpine D and Johnstone BM (1990) The ototoxic mechanism of cisplatin. *Hear Res* **47**: 191–204

Melamed LB, Selim MA, Facs F and Schuchman D (1985) Cisplatin ototoxicity in gynecologic cancer patients. *Cancer* **55**: 41–43

Moul JW, Robertson JE, George SL, Paulson DF and Walther PJ (1989) Complications of chemotherapy for testicular cancer. *J Urol* **142**: 1491–1496

Nakai Y, Konishi K, Chang KC, Ohashi K, Moriaski N, Minowa Y and Morimoto A (1982) Ototoxicity of the anticancer drug cisplatin. *Acta Otolaryngol* **93**: 227–232

Osanto S, Bukman A, Van Hoek F, Sterk PJ, De Laat JAPM and Hermans J (1992) Long-term effects of chemotherapy in patients with testicular cancer. *J Clin Oncol* **10**: 574–579

Piel IJ, Meyer D, Perlia CP and Wolfe VI (1974) Effects of cis-diamminedichloroplatinum (NSC-119875) on hearing function in man. *Cancer Chemother Rep* **58**: 871–875

© Cancer Research Campaign 1998

Ravi R, Somani SM and Rybak LP (1995) Mechanism of cisplatin ototoxicity: antioxidant system. *Pharmacol Toxicol* **76**: 386–394

Reddel RR, Kefford RF, Grant JM, Coates AS, Fox RM and Tattersall MHN (1982) Ototoxicity in patients receiving cisplatin: importance of dose and method of drug administration. *Cancer* **66**: 19–23

Riggs LC, Brummett RE, Guitjens SK and Matz GJ (1996). Ototoxicity resulting from combined administration of cisplatin and gentamycin. *Laryngoscope* **106**: 401–406

Rybak LP, Ravi R and Somani SM (1995) Mechanisms of protection by diethyldithiocarbamate against cisplatin ototoxicity: antioxidant system. *Fundam Appl Toxicol* **26**: 293–300

Saito T and Aran JM (1994) Comparative ototoxicity of cisplatin during acute and chronic treatment. *ORL J* **56**: 315–320

Schaefer SD, Post JD, Close LG and Wright CG (1985) Ototoxicity of low- and moderate-dose cisplatin. *Cancer* **56**: 1934–1939

Schilsky RL (1982) Renal and metabolic toxicities of cancer chemotherapy. *Semin Oncol* **9**: 75–81

Schwabe H-R, Herrmann R, Mathew M, Graef K-J, Sander T, Cordes M, Nagel R, Weissbach L and Huhn D (1992) Langfristige Toxizität der Polychemotherapie bei kurativ behandeltem Hodenkarzinom. *Dtsch Med Wschr* **117**: 121–126

Schweitzer VG (1993) Ototoxicity of chemotherapeutic agents. *Otolaryngol Clin North Am* **26**: 759–789

Skinner R, Pearson ADJ, Amineddine HA, Mathias DB and Craft AW (1990) Ototoxicity of cisplatin in children and adolescents. *Br J Cancer* **61**: 927–931

Stoter G, Koopman A, Vendrik CPJ, Struyvenberg A, Sleyfer DT, Willemse PHB, Schraffordt Koops H, Van Oosterom AT, Ten Bokkel Huinink WW and Pinedo HM (1989) Ten-year survival and late sequelae in testicular cancer patients treated with cisplatin, vinblastine, and bleomycin. *J Clin Oncol* **7**: 1099–1104

Stuart NSA, Woodroffe CM, Grundy R and Cullen MH (1990) Long-term toxicity of chemotherapy for testicular cancer – the cost of cure. *Br J Cancer* **61**: 479–484

Van Der Hulst RJAM, Dreshler WA and Urbanus NAM (1988) High-frequency audiometry in prospective clinical research of ototoxicity due to platinum derivates. *Ann Otol Rhinol Laryngol* **97**: 133–137

Vermorken JB, Kapteijn TS, Hart AAM and Pinedo HM (1983) Ototoxicity of cis-diamminedichloroplatinum (II): influence of dose, schedule and mode of administration. *Eur J Cancer Clin Oncol* **19**: 53–58

Waters GS, Ahmad M, Katsarkas A, Stanimir G and McKay J (1991) Ototoxicity due to cis-diamminedichloroplatinum in the treatment of ovarian cancer: influence of dosage and schedule of administration. *Ear Hear* **12**: 91–102

© Cancer Research Campaign 1998